# Quaternary history, population genetic structure and diversity of the cold-adapted Alpine newt *Ichthyosaura alpestris* in peninsular Italy

**DOI:** 10.1038/s41598-017-03116-x

**Published:** 2017-06-07

**Authors:** Andrea Chiocchio, Roberta Bisconti, Mauro Zampiglia, Giuseppe Nascetti, Daniele Canestrelli

**Affiliations:** Dipartimento di Scienze Ecologiche e Biologiche, Università della Tuscia, Viale dell’Università s.n.c., I-01100 Viterbo, Italy

## Abstract

Mediterranean peninsulas are major biodiversity hotspots, and cold-adapted species are an important component of this biodiversity. However, cold-adapted species contributed surprisingly little to our knowledge of the intimate links between Quaternary environmental changes, species’ responses to these changes, and current patterns of intraspecific biodiversity. Here, we investigated the genetic structure and evolutionary history of a cold-adapted amphibian, the Alpine newt *Ichthyosaura alpestris*, within the Italian peninsula. Nuclear and mitochondrial markers consistently identified three distinct genetic lineages, whose divergence dates to the Early Pleistocene (1.9 and 0.8 million years ago). Our results show that the Italian peninsula provided multiple Pleistocene refugia to this cold-adapted species, and suggest that allopatric fragmentation followed by secondary admixture have been key events in the formation of its current pattern of genetic diversity. Indeed, estimates of population genetic diversity clearly identified contact populations as those achieving the highest levels of diversity. Such concordance among cold-adapted and temperate species in terms of processes triggering the formation of regional patterns of genetic diversity provides strong support for the hypothesis that gene exchange between divergent lineages, rather than long-term stability of refugial populations, has been the main step toward the formation of hotspots of intraspecific biodiversity.

## Introduction

Understanding how past climatic oscillations, topographic features and species-specific ecological characteristics have contributed to shape spatial and temporal patterns of intraspecific genetic variation has been a major goal of the phylogeographic exploration since its inception^1–3^, and the Mediterranean peninsulas have been iconic regions in this exploration^4,5^. After over three decades of investigating phylogeographic patterns in temperate species^5–7^, it is now well established that Pleistocene climatic oscillations have played a prominent role in this regard. By promoting cycles of contraction and expansion in species’ ranges, they triggered the formation of range-wide patterns of genetic diversity, such as the so-called “southern richness and northern purity” pattern^8–11^. Moreover, through their interactions with the topographical complexity of the Mediterranean peninsulas and their influence on sea-level changes, Pleistocene climatic oscillations also contributed to the formation of complex patterns of population genetic structure and divergence within these regions, such as the so-called “refugia-within-refugia”^12^. Most of the microevolutionary processes that prompted the formation of current hotspots, cold spots, and melting pots of genetic diversity were triggered by interactions between climatic oscillations and topographical complexity^12–18^. Accordingly, despite differences among species that are related to their ecological characteristics, genetic imprints of Pleistocene climatic oscillations have been observed in virtually all of the temperate species studied to date (but see Porretta *et al*.^19^). Biological diversity within Mediterranean peninsulas, however, is not restricted to temperate species.

Mountain areas are widespread within these peninsulas, and disproportionately contribute to habitat heterogeneity^20^. Although their role in the evolutionary history of local biota has not been overlooked, they have mainly been characterized as barriers to dispersal for temperate species. However, mountain areas also contain a wealth of cold-adapted species^21^, whose contribution to peninsular biodiversity is not negligible^20,22,23^ and that are expected to be particularly sensitive to climatic oscillations^21,24^. Several observations suggest that the responses of cold-adapted and temperate species to Pleistocene climate changes differed considerably. In southern Europe, climatic optima of cold-adapted species are mainly arranged along steep altitudinal clines within strongly discontinuous mountain ranges, which *per se* form the basis for population fragmentation. However, interglacial phases were shorter than glacial phases, suggesting that there were shorter time periods for cold-adapted species to accumulate substantial differentiation among populations^18,21,24–28^. Therefore, the large amount of data available for temperate species appears to provide only a limited foundation for predictions about cold-adapted species’ responses to climatic oscillations. At the same time, the evolutionary histories of cold-adapted species in response to Pleistocene climate changes within Mediterranean peninsulas are still largely understudied. This paucity of phylogeographical studies is particularly true of the Italian peninsula. This peninsula is crossed throughout its length by the Apennine, a mid-to-high-altitude mountain chain that runs for over 1200 km along a NW-SE direction. The Apennines contain a wealth of cold-adapted taxa that often exhibit disjointed and relict-like distributions. Despite this, a literature search using combinations of the terms Phylogeography, Italian/Italy, and Apennines (ISI Web of Science; last accessed 24^th^ August 2016) retrieved 43 papers dealing with the phylogeographical structure of populations within the Italian peninsula, but only six papers concerned cold-adapted species^29–34^ (see Discussion). Furthermore, the few studies that have been conducted were based on a limited sampling of the Apennine populations and/or on the use of only mitochondrial DNA as a molecular marker, as was customary until recently.

**Figure 1:**
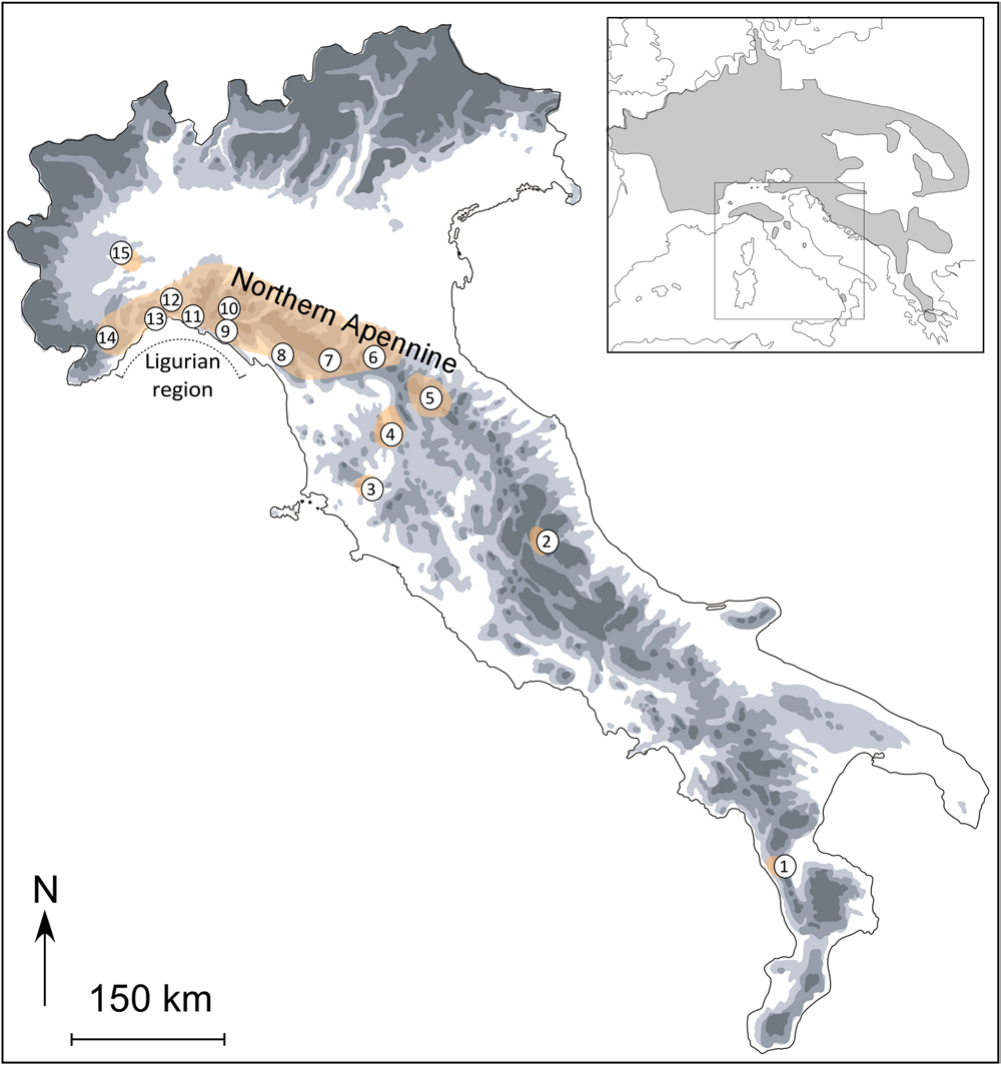
Geographical distribution of *Ichthyosaura alpestris* on the Apennine peninsula and the geographical locations of the 15 populations sampled. The locations are numbered as in Table 1. The inset shows the distribution of *I*. *alpestris* within the Western Palearctic region. The map was drawn using the software Canvas 11 (ACD Systems of America, Inc.).

In this study, we investigated the genetic structure and evolutionary history of a cold-adapted amphibian, the Alpine newt *Ichthyosaura alpestris* (Laurenti, 1768), within the Italian peninsula. This species is widely distributed in central and eastern Europe, while in the Apennines its distribution is fragmented and closely linked to mid- and high-altitude ponds^35^ (Fig. 1). Morphological and ecological data suggest that the Apennine populations belong to two distinct subspecies: *I*. *a*. *apuana* in the northern and central Apennines and *I*. *a*. *inexpectata* in the southernmost, isolated Calabrian region^36–38^. Phylogenetic analyses have confirmed that populations in the Italian peninsula belong to a distinct evolutionary unit, and have estimated their divergence from other European clades (including populations in the Alps) to the Late Miocene^39–41^.

Here, using information from both mitochondrial and nuclear markers in combination with population genetic, phylogenetic and Bayesian phylogeographical tools, we aimed to assess (1) the current patterns of genetic diversity of the Alpine newt within the Italian peninsula, (2) the geographical structure of genetic variation among its populations, and (3) its Pleistocene evolutionary history. To the best of our knowledge, this is among the few studies that have combined exhaustive sampling with a multi-locus dataset to investigate the Pleistocene evolutionary history and genetic diversity of a cold-adapted animal species within Mediterranean peninsulas, and the first one for the Italian peninsula.

## Results

### Patterns of genetic variation

We sequenced two mitochondrial DNA (mtDNA) fragments from 149 alpine newt individuals: a 399-bp fragment of the Cytochrome B gene (*CytB*) and an 878-bp fragment of mitochondrial NADH dehydrogenase subunit 2 gene (*ND2*). No indels, stop codons or non-sense codons were observed on either gene. In the combined mtDNA dataset (1277 bp), we found 13 haplotypes defined by 35 variable positions, 31 of which were parsimony informative. The mean haplotype diversity (*h*) and nucleotide diversity (π) values for this dataset were 0.803 (±0.019 SD) and 0.0077 (±0.0002 SD), respectively.

The nuclear DNA (nDNA) dataset included a 328-bp fragment of the β-fibrinogen gene (*β*-*FIB*) from 104 individuals, a 565-bp fragment of the platelet-derived growth-factor receptor gene (*PDGFR*) from 111 individuals, and a 670-bp fragment of the growth hormone gene (*GH*) from 103 individuals. In the *β*-*FIB* alignments, we found six haplotypes that were identified by eight variable sites, seven of which were parsimony informative; *h* = 0.365 (±0.041 SD) and π = 0.0041 (±0.001 SD). In the *PDGFR* alignments, we found five haplotypes that were identified by four variable sites, all parsimony informative; *h* = 0.413 (±0.038 SD) and π = 0.0018 (±0.0002 SD). In the *GH* alignments, we found eight haplotypes that were identified by 17 variable sites, 15 of which were parsimony informative; *h* = 0.376 (±0.039 SD) and π = 0.0017 (±0.0004 SD). No recombination events were indicated by the PHI tests conducted on the nuclear gene fragments (all *P* > 0.05). Using phased haplotypes as alleles, a multilocus genotype matrix was built that included 121 individuals for the three nuclear loci, with 8% of missing data. A full list of all of the haplotypes found for each gene in each sampled population is presented as Supplementary Table S1.

A total of 185 individuals were genotyped at the nine microsatellite loci. *TaCa1* exhibited no variation across the samples and was excluded from further analyses. *Ta2Caga3* was also removed, because it tested positive for null alleles in four out of fifteen populations. The final dataset consisted of a multilocus genotype for 185 individuals at seven microsatellite loci, with 2.8% of missing data. We found a significant deviation from the Hardy-Weinberg equilibrium in population 13 for *Copta9*, although it was not significant after the Bonferroni correction was applied. No linkage disequilibria were found. Across all populations, the number of alleles at each locus ranged from 2 (*Copta 13* and *Copta 1*) to 19 (*Copta 9*). Allele frequency data for each locus in each population are given as Supplementary Table S1, and allelic richness and mean expected heterozygosity estimates for each population are presented in Table 1. The northernmost and isolated population 15 was the one showing the lowest values of genetic diversity, whereas the highest values were observed for the north-western population 9.

**Table 1:** Geographical locations of the 15 *Ichthyosaura alpestris* sampling sites, sample sizes for each gene studied, and estimates of genetic variability at microsatellite loci for each population.

	Locality	Lat. N	Long. E	mtDNA	β-FIB	PDGFR	GH	Microsatellites		
								n	*Ar*	*He*
1	Montalto Uffugo	39° 33′	16° 01′	11	6	9	9	14	1.99	0.22 (0.34)
2	Monti della Laga	42° 42′	13° 19′	10	7	7	7	14	2.13	0.25 (0.36)
3	Iesa	43° 05′	11° 14′	4	4	4	4	7	2.17	0.28 (0.36)
4	Torsoli	43° 33′	11° 23′	9	9	8	7	12	1.97	0.19 (0.32)
5	Camaldoli	43° 48′	11° 49′	11	6	6	6	13	2.38	0.30 (0.35)
6	Monghidoro	44° 13′	11° 17′	7	8	5	7	10	2.27	0.28 (0.36)
7	Lago del Greppo	44° 07′	10° 40′	9	11	10	10	12	2.36	0.24 (0.34)
8	Minucciano	44° 09′	10° 14′	10	8	8	8	13	1.87	0.23 (0.31)
9	Stagno Bargone	44° 19′	09° 29′	14	11	14	8	16	2.51	0.40 (0.32)
10	Monte Penna	44° 29′	09° 29′	14	8	9	7	14	2.44	0.38 (0.32)
11	Capanne di Marcarolo	44° 33′	08° 46′	9	5	6	5	14	2.47	0.37 (0.38)
12	Rossiglione	44° 32′	08° 37′	9	5	5	4	10	2.05	0.26 (0.31)
13	Piampaludo	44° 26′	08° 35′	12	6	8	7	13	2.50	0.32 (0.36)
14	Madonna del Lago	44° 07′	07° 59′	10	5	5	6	13	1.75	0.21 (0.31)
15	Pecetto Torinese	45° 02′	07° 43′	10	5	7	8	10	1.43	0.15 (0.27)

n, sample size; Ar, allelic richness; He, expected heterozygosity (with standard deviation).

### Population genetic structure

**Figure 2:**
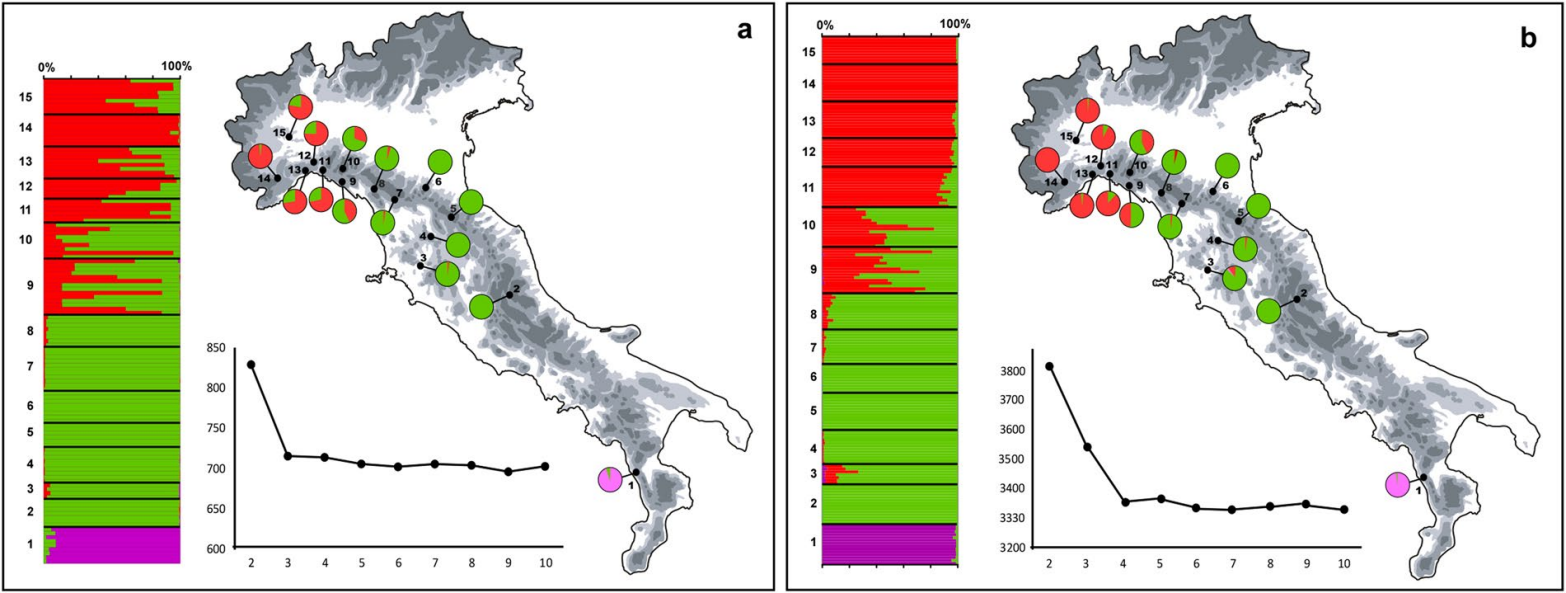
Genetic structure of *Ichthyosaura alpestris* populations in Italy estimated using TESS based on (**a**) nuclear sequence dataset and (**b**) a microsatellite dataset. The bar plots show the admixture proportions of each individual for the three genetic clusters recovered. The pie diagrams on the maps show the frequency distributions of each cluster among the populations. The line charts show mean values of deviance information criterion (DIC) statistics (averaged over 100 runs) that were estimated for the models, with the number of genetic clusters (K) ranging from 2 to 10. The map was drawn using the software Canvas 11 (ACD Systems of America, Inc.).

The Bayesian clustering analyses conducted with TESS clearly and consistently showed that the population genetic structure of the Alpine newt within the Italian peninsula is best represented by three distinct clusters. Indeed, K = 3 was the best clustering option for the nDNA dataset, because only a minor decrease in the deviance information criterion (DIC) values was observed at higher K values (Fig. 2a). DIC values with the microsatellite dataset reached a plateau at K = 4 (Fig. 2b), but the inspection of the plotted membership coefficients showed that only three clusters were represented (see also refs^42, 43^).

The spatial distribution of the three clusters had a clear geographical structure: one cluster was mainly restricted to the north-western populations (9–15), one was by far the most represented among populations in the north-central Apennines (2–10), and the third was geographically restricted to the southernmost population (1). Bar plots showing the individual admixture proportions and pie-charts showing the average proportion of each cluster within each sampled population are presented in Fig. 2. The geographically intermediate populations 9 and 10 were the most admixed, because all of the individuals were of mixed ancestry; admixture proportions gradually decreased away from these populations. In general, the nDNA data revealed higher admixture proportions than the microsatellite data, particularly among north-western populations.

The analysis of molecular variance (AMOVA) analysis was performed with the following groupings: [N-W clade: samples 11–15], [S-E clade: samples 2–10], [Calabrian: sample 1]. With this grouping, 24.6% of variation was attributed to the among-group level of variation (F_CT_: 0.25), 14.5% to the among-population within groups level (F_SC_: 0.19), 60.9% to the within population level (F_ST_: 0.39); all variance components and fixation indices were statistically significant (P < 0.001).

### Phylogenetic and Bayesian phylogeographical analyses

**Figure 3:**
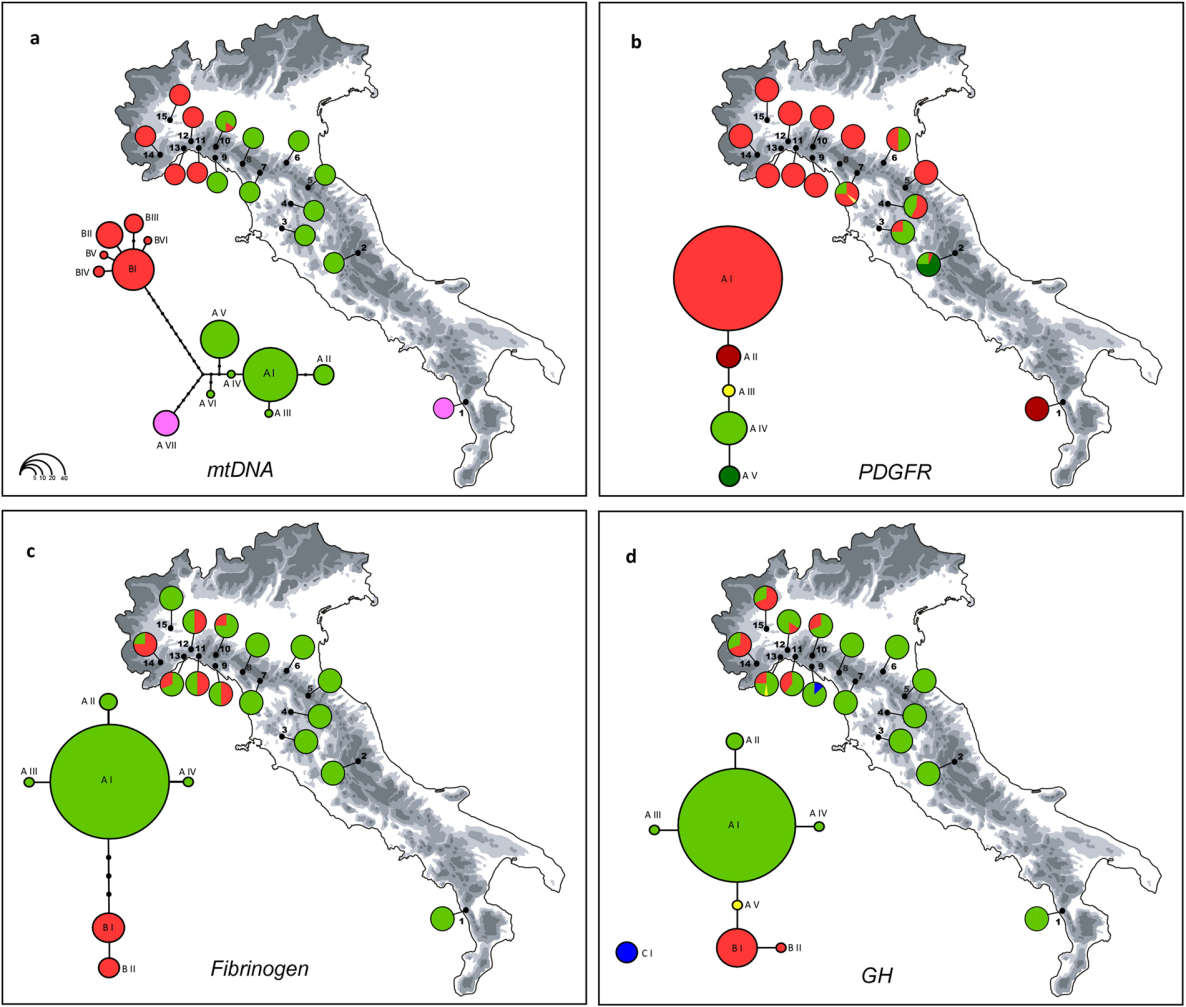
Haplotype genealogy based on a maximum likelihood phylogenetic tree of the *Ichthyosaura alpestris* mtDNA haplotypes found (**a**). Statistical parsimony network showing genealogical relationships among haplotypes in *PDGFR* (**b**), *β*-*FIB* (**c**) and *GH* (**d**) genes. Circle sizes are proportional to haplotype frequency; missing intermediate haplotypes are shown as black dots. Pie diagrams show the frequency distributions of the haplogroups among the populations studied. The map was drawn using the software Canvas 11 (ACD Systems of America, Inc.).

The phylogenetic network of the mtDNA haplotypes yielded by Haplotype Viewer is shown in Fig. 3a. The log-likelihood score for the ML tree was −1963.08. Three main haplogroups were found, with a geographical distribution that mirrored the Bayesian clustering of multilocus genotypes: a north-western Apennine haplogroup (samples 10–15; hereafter NW haplogroup), a north-central Apennine haplogroup (samples 2–10; hereafter CA haplogroup) and a Calabrian haplogroup made up of a single haplotype, which was closely related to the north-central Apennine haplogroup and was found exclusively in the southernmost population (sample 1). The geographically intermediate population of Mount Penna (sample 10) was the only one in which haplogroups NW and CA co-occurred. The statistical parsimony analysis retrieved the same topology as the ML analysis, but two distinct networks were generated, one for the NW haplogroup and one for the CA and Calabrian haplogroups (data not shown). The net sequence divergence among NW and the other haplogroups was 0.012 (±0.003 SE), while between CA and the Calabrian haplogroup it was 0.007 (±0.002 SE).

The phylogenetic networks among haplotypes at each nuclear gene fragment are given in Fig. 3. In all cases, one single network connected all of the haplotypes, except for one haplotype of the *GH* gene fragment that did not connect with the main network. Although all three genes exhibited very low levels of variation, a geographical structure was apparent, with distinct haplotype frequencies observed among north-western, north-central and, (at the sole *PDGFR*) southern populations (see Fig. 3 and Supplementary Table S1).

**Figure 4:**
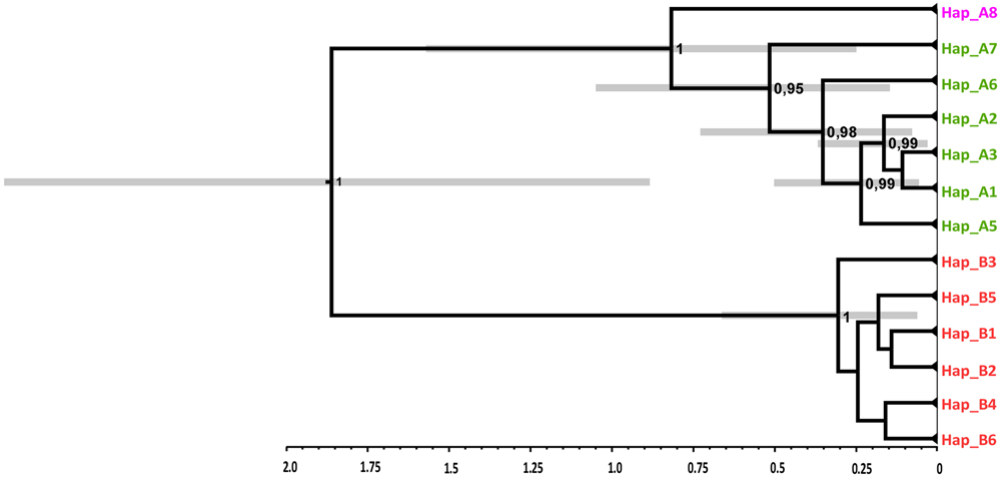
Maximum clade credibility tree based on the *Ichthyosaura alpestris* mtDNA dataset recovered by Bayesian analysis in BEAST, showing the time to the most recent common ancestor (TMRCA) for the major clades. Node bars (grey) represent 95% highest posterior density (HPD) intervals for node ages. The posterior probabilities for each node are also shown (only values >0.9 are shown). The scale is in millions of years before present. The graph was drawn using the software Canvas 11 (ACD Systems of America, Inc.).

The divergence time estimation among mtDNA haplogroups yielded by BEAST runs fully converged to a stationary distribution, with high effective sample size (ESS) values (>200) for all the parameters of interest. The substitution rate was estimated as 4.586 × 10^−9^ substitutions/site/year [95% highest posterior density (HPD) interval between 1.766 × 10^−9^ and 8.08 × 10^−9^]. A chronogram based on the maximum clade credibility (MCC) tree is presented in Fig. 4. The tree topology was consistent with the phylogenetic network analyses, as it showed a deep and ancient split between the NW haplogroup and the other haplotypes, which was estimated to have occurred 1.86 million years ago (mya) (95% HPD, 0.89–2.86), followed by a more recent split between the CA and Calabrian haplogroups (mean 0.82 mya; 95% HPD, 0.25–1.57). The times to the most recent common ancestor (TMRCA) of haplogroups NW and CA were estimated at 306 000 (95% HPD, 65 000–661 000) and 514 000 (95% HPD, 157 000–1 041 000) years, respectively.

**Figure 5:**
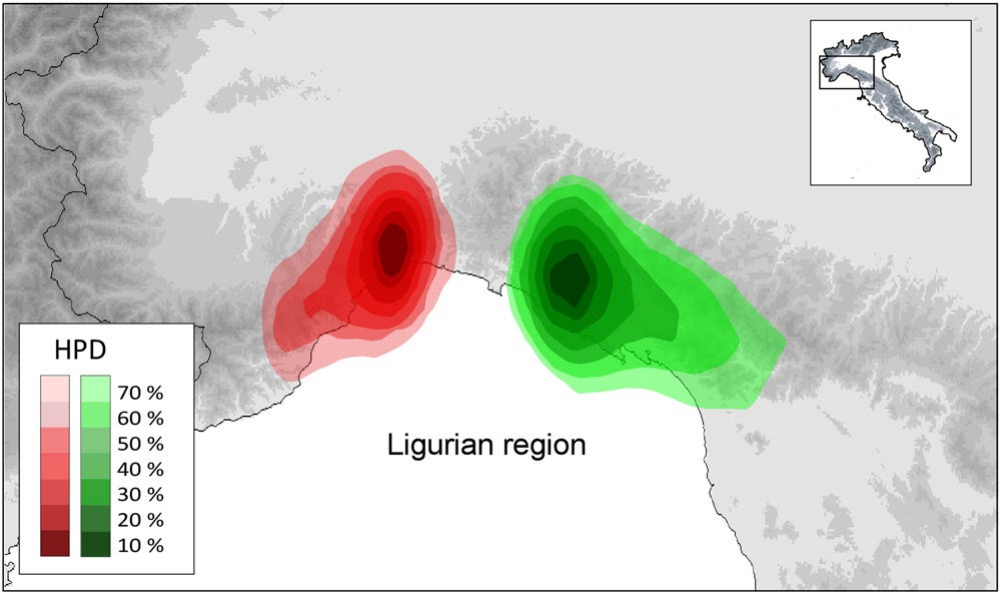
Ancestral areas of the two main genetic lineages of *Ichthyosaura alpestris* at their respective times to the most recent common ancestor (TMRCAs) based on Bayesian phylogeographical analyses conducted independently for both lineages on mtDNA. Polygons represent the highest posterior density (HPD) regions of the geographical locations at 10% to 70% probabilities. The map was drawn using the software Canvas 11 (ACD Systems of America, Inc.).

Bayesian phylogeographical analyses were conducted for the mtDNA lineages NW and CA separately, whereas the Calabrian lineage was excluded from this analysis because it lacked variation. For each lineage, the two runs converged to a stationary distribution and had satisfactory (>200) ESS values. The ancestral areas of these lineages (at their respective TMRCAs) were estimated to have occurred in close geographical contiguity, along the north-western part of the *I*. *a*. *apuana* range (see Fig. 5). However, spatial diffusion from these areas occurred earlier for CA than for NW (see Supplementary Information .kmz file). Indeed, CA almost completed its spatial diffusion before the last interglacial phase (approximately 125 000 years ago), with the possible exception of the southernmost, isolated population of Monti della Laga (sample 2), which was probably established soon after the last glacial maximum (18 000 years ago). In contrast, the spatial diffusion of NW occurred entirely during the last glacial phase, when secondary contact between both lineages was established.

## Discussion

Mitochondrial and nuclear markers consistently supported the existence of three strongly differentiated lineages of *I*. *alpestris* on the Apennine peninsula^40,41^ one restricted to the southernmost and isolated populations in Calabria, one ranging from the northern to the central Apennines, and one ranging from the western to the eastern Ligurian Apennines.

The Calabrian populations are over 500 km from the nearest population in the central Apennines, and exhibit genetic divergence of a likely mid-Pleistocene origin. The low level of genetic diversity found in this area was not unexpected, because population size was estimated to be extremely low (only about 300 individuals^44^). Despite a substantial divergence, the other two lineages exhibited a contiguous distribution with a contact zone occurring in the eastern Ligurian Apennines, where the populations are strongly admixed. The contact zone was wider for nuclear than mitochondrial genes, as only one population shared the mitochondrial haplotypes of both lineages. This type of mito-nuclear discordance can be accounted for by male-biased dispersal^45^, which can result in a more rapid dispersal of nuclear genes (biparental) than mitochondrial genes (only matrilineal). Male-biased dispersal has been reported in juvenile *I*. *alpestris*^46^, and has previously been invoked to explain mito-nuclear discordance in this species^41^. Male-biased dispersal could have increased due to the high prevalence of paedomorphic females in the Apennine^47^, which do not disperse from natal populations^48^.

The contact zone observed among the Alpine newt lineages within the Ligurian Apennines falls within a ‘crossroad’ area of recolonization routes for species that altered their ranges to cope with Pleistocene climate changes^3,9^. Moreover, secondary contacts between closely related lineages have previously been observed within this area (e.g. refs^49, 50^), and it marks the range boundary of several other taxa, including amphibians (e.g. *Hyla meridionalis*, *Pelodytes punctatus*, and *Rana italica*). Such a clustering of contact zones, recolonization routes, and biogeographical breaks suggests that this area might be a suture zone (*sensu* Remington^51^)^8,52^. Whether common patterns among species and intraspecific lineages occur by chance or are the outcome of shared historical processes is an open question that requires further investigation.

In principle, the peculiar climatic and/or environmental features of this area might have promoted its role as a barrier to dispersal along an east-west axis. However, obvious geographical discontinuities are not apparent in this area. On the other hand, the Ligurian Apennines are particularly close to the sea in this area, which has major consequences for the local climate, and climatic discontinuities between the western and eastern Ligurian Apennines have been identified both for temperature and rainfall^53^.

The strong genetic differentiation found among the Apennine lineages of *I*. *alpestris*, and among them and other European lineages, suggests that the occurrence of the Alpine newt on the Apennine peninsula is of ancient origin. Recuero *et al*.^41^ estimated the divergence among Apennine and other European lineages to about 9.2 ± 2.1 mya, suggesting that *I*. *alpestris* might have colonized the Apennine peninsula during the Late Miocene (but see ref.^40^ for an alternative dating). Subsequently, the evolutionary history of *I*. *alpestris* on the Apennine peninsula seems to have been strongly affected by the climatic revolutions of the Pleistocene.

The divergence between north-western Apennine and north-central Apennine lineages was estimated to have occurred during the Early Pleistocene (1.86 mya ± 1.0), which coincided with the onset of the major glacial-interglacial cycles^54^. The general cooling of climatic conditions induced by early glaciations promoted the spread of suitable habitats for frigophilous species, such as *I*. *alpestris*, at lower altitudes and latitudes^55^, likely triggering range expansions along the peninsula. A general diffusion of other cold-adapted taxa during the Early Pleistocene has also been reported by refs^54–56^. On the other hand, warm interglacials might have fragmented the distributions of these species after their early expansions, trapping populations within small areas, and promoting their differentiation.

The mean values of the TMRCA for both the Calabrian and the central Apennine lineages were estimated at about 800 000 years ago, i.e. close to the so-called Mid-Pleistocene revolution^57^. The amplitude and duration of the glacial cycles substantially increased during this epoch, with major biogeographical effects^5^. Along the Italian peninsula, major changes in community assemblages occurred^58,59^, and several species’ ranges underwent severe fragmentation followed by genetic differentiation^60,61^. Noteworthy, two particularly humid glacial cycles [corresponding to Marine Isotope Stage (MIS) 20 and MIS 18, which occurred 750–850 000 years ago] caused the expansion of Alpine forests in Calabria^58^, which probably promoted a southward expansion of *I*. *alpestris* and the consequent establishment of the Calabrian population. Such a large expansion may have been a unique event, as evidenced by the strong differentiation between lineages, and by the lack of evidence in our data for successive contacts and admixture between them.

In contrast, the secondary contact between the north-western and north-central Apennine lineages was estimated to have occurred during the last glacial phase, and was driven by an eastward expansion of the former. However, our spatio-temporal phylogeographical reconstruction revealed that most of the expansion from the north-central Apennine refugia had already occurred at the end of the penultimate glacial period (MIS 6: 185–135 000 years ago^62^), and was completed during the last glacial phase. This scenario supports the hypothesis that *I*. *alpestris* populations underwent major expansions during cold, glacial phases. Noteworthy, the penultimate glacial phase was particularly cold and humid, and was interspersed by several interstadials with intermediate climatic conditions^62–64^. Such unusual climatic conditions promoted the expansion of montane forests in southern Europe^62^, and could also have favoured a greater expansion of *I*. *alpestris* from its refugia.

Haplotype diversity within each group and population was low compared to that of other peninsular amphibians (i.e. *Lissotriton italicus*^65^, *Triturus carnifex*^60^ and *L*. *vulgaris*^61^), but was similar to that observed within other cold-adapted amphibians, such as *Rana temporaria*^32^. In addition, the population genetic diversity as estimated by the microsatellite markers was lower than that of other European populations of the same species^66–68^. These low levels of genetic diversity could have been caused by repeated bottlenecks of the Apennine populations during the Pleistocene, which were caused by the fragmentation and isolation of the peninsular populations. The lowest values of genetic diversity were observed within both the southernmost and the northernmost populations (samples 1 and 15). Low levels of genetic diversity are commonly observed in peripheral populations^69^, as a result of both historical and recent processes^70^. Our phylogeographic reconstruction suggests that the observed paucity of genetic diversity in the northernmost population is linked to its recent foundation, whereas in the southernmost population it could have been caused by ancient isolation. On the contrary, genetic diversity increased towards the centre of the species’ range in the Apennines. Interestingly, the highest levels of genetic diversity were found within the populations closest to the contact zone, where all of the individuals were highly admixed. This pattern closely parallels similar observations in temperate taxa, in which the highest levels of genetic diversity have been found within zones of admixture among previously allopatric lineages, rather than within populations from the inferred refugia^17,71^. In turn, this does not support the long-invoked hypothesis that the formation of hotspots of genetic diversity has been promoted by the prolonged stability of populations within refugia^72,73^. Instead, it highlights the importance of sub-refugia in shaping genetic variation, and of melting-pot areas in reshuffling such variation and promoting the formation of hotspots^13^.

## Conclusions

As previously observed in different Mediterranean regions (including both continental and insular contexts, see e.g. refs^5,12,74–79^), Pleistocene refugia for cold-adapted species within the Italian peninsula do not match those identified for most temperate species. Indeed, while refugia for temperate species have been mainly found in the southern part of the Italian peninsula^17,60,65,71,80,81^, refugia for cold-adapted species have been identified in the northwest (as in the case of the Alpine newt) and elsewhere in the northern half of the Apennine chain^15,21,29–34^. Moreover, glacial phases had the opposite effect on cold-adapted taxa than on temperate taxa, because they promoted range expansions rather than contractions. Nevertheless, despite such differences, common patterns are emerging, that are shared by temperate and cold-adapted species. Refugial ranges were often fragmented, promoting genetic differentiation within the peninsular region. The areas hosting populations with the highest levels of genetic diversity do not strictly overlap with areas of long-term demographic stability (i.e. refugia). Instead, hotspots of genetic diversity are often found in areas of admixture between intraspecific genetic lineages, which have survived periods of unfavourable climatic conditions within separate sub-refugia.

Such concordance among cold-adapted and temperate species in terms of the processes that trigger the formation of range-wide patterns of genetic diversity provides strong support for the hypothesis that gene exchange between divergent lineages, rather than long-term stability, is the main driver for the formation of hotspots of intraspecific biodiversity.

## Methods

### Sampling, laboratory procedures, and descriptive statistics

We collected 185 *I*. *alpestris* individuals from 15 localities that spanned its distribution in the Apennines; sampling locations and sample sizes are given in Table 1 and Fig. 1. Tissue samples were collected from tail tips after the newts had been anaesthetized by submersion in a 0.1% solution of MS222 (3-aminobenzoic acid ethyl ester). All of the individuals were then released at their respective collection sites. Sampling activities and the tail-clipping procedure for tissue collection were approved by the Italian Ministry of Environment (permit number: DPN-2009-0026530) and were performed in accordance with relevant guidelines and regulations. Samples were stored in 95% ethanol until subsequent analyses.

DNA extractions were performed following the standard cetyltrimethylammonium-bromide (CTAB) protocol^82^. Two mtDNA and three nDNA fragments were amplified and sequenced. The mtDNA fragments were from the *CytB* gene and from the *ND2* gene; the three nDNA fragments were from the fourth intron of the *GH* gene, the *β*-*FIB* gene, and the *PDGFR* gene. The polymerase chain reaction (PCR) primers used, their sequences and their annealing conditions are presented in Table 2. Amplifications were performed in a 10-μL reaction volume containing MgCl_2_ (2 mM), four dNTPs (0.2 mM each), two primers (0.2 μM each), the enzyme *Taq* polymerase (0.5 U, Promega), its reaction buffer (1X, Promega) and 40–200 ng of DNA template. The PCRs were conducted with an initial step at 95 °C for 5 min, 32 cycles at 94 °C for 1 min, 40″ of annealing (see Table 2), 72 °C for 1 min, followed by a single final step at 72 °C for 5 min. To increase the specificity and yield of the *β*-*FIB* amplification, we used a nested PCR as in Sequeira *et al*.^83^, with slight modifications in the cycling conditions of the second PCR (26 cycles at 94 °C for 30″, 59 °C for 30″ and 72 °C for 45″). The purification and sequencing of the PCR products were conducted on both strands by Macrogen Inc. (http://www.macrogen.com), using an ABI PRISM^®^ 3730 sequencing system (Applied Biosystems). All of the sequences were deposited in GenBank (accession numbers: KY911407–KY911451).

**Table 2:** Polymerase chain reaction primers and annealing temperatures (°C) used to amplify the two mitochondrial and three nuclear DNA fragments that were used in this study.

Marker	Primers (5′ -> 3′)		T	Ref.
CytB	CytBtrit-F	ACGCAAYATRCACATCAACGG	53	39
	CytBtrit-R	GGAGTGACTATAGARTTTGCTGGG		39
NADH2	L3780mod2	GGAGAAACCCCTTCTTTTGC	59	This study
	H5018mod1	TGAAGGCCTTTGGTCTTGTTAT		This study
GH	GH-f	TCTCATCAAGGTGAGTTTGAACA	58	101
	GH-r	CCTTCTTGTGTCAGAGGTGCTAT		101
PDGF-R	Pdgfr-F	TGCAGCTGCCATATGACTCTA	60	101
	Pdgfr-R	TACGCTGTTCCTTCAACCACT		101
β-FIB	FibX7	GGAGANAACAGNACNATGACAATNCAC	50	75
	FibX8	ATCTNCCATTAGGNTTGGCTGCATGGC		75
	BFXF	CAGYACTTTYGAYAGAGACAAYGATGG	59	75
	BFXR	TTGTACCACCAKCCACCRTCTTC		75

We also analysed genetic variation at the level of nine microsatellite loci (*Copta1*, *Copta3*, *Copta8*, *Copta13*, *Copta9*, *TaCa1*, *Ta3Ca8*, *Ta3Caga2*, and *Ta2Caga3*), following previously published protocols^67,84,85^. We chose a subset of available loci after excluding those that exhibited reaction inconsistency in over 30% of samples. Forward primers were fluorescently labelled and PCR products were electrophoresed by Macrogen Inc. on an ABI 3730xl genetic analyser (Applied Biosystems) with a 400-HD-size standard.

Electropherograms of sequence data were visually checked using FinchTV 1.4.0 (Geospiza Inc.), and sequences were aligned using Clustal X 2.0. Heterozygous nuclear sequences were phased using the PHASE method^86^ in DnaSP 5.10 using default parameter values; superimposed traces produced by heterozygous indels were resolved following Flot *et al*.^87^. For each nuclear gene, the probability of recombination was evaluated using the pairwise homoplasy index (PHI statistics^88^) in SplitsTree 4.13.1. All of the subsequent analyses were conducted using phased nuclear data, and indels were treated as missing data. Nucleotide variation was assessed using MEGA 6.0; haplotype and nucleotide diversity were estimated using DnaSP 5.10.

The microsatellite data were analysed using GeneMapper^®^ 4.1. Micro-Checker 2.2.3 was used to test for null alleles and large-allele dropout influences. Because the tetranucleotide locus *Copta9* exhibits a dinucleotide variation in some populations, each allele was sequenced as above; sequence alignment revealed a two-base-pair insertion in the flanking region of the simple sequence repeat (SSR) of some alleles. To use only true SSR polymorphisms in our analyses, the fragment-length values of these alleles were corrected by subtracting 2. Allelic frequencies and estimates of genetic diversity were based on the mean allelic richness and the mean observed and expected heterozygosity, and we tested for deviations from the expected Hardy-Weinberg and linkage equilibria using the programs GENETIX 4.05 and FSTAT 2.9.3.

### Population genetic structure

The population genetic structure across the study area was evaluated using the microsatellite and nuclear sequence datasets separately, in order to check for consistency among marker types and to keep the amount of incomplete multilocus genotypes to a minimum. We used the Bayesian clustering algorithm implemented in TESS 2.3.1^89^ with the actual geographical distributions of individuals as prior information, because it performs better than other Bayesian methods with shallow genetic structures and a limited number of loci^89,90^. For the nuclear sequences dataset, we compiled a multilocus genotype data matrix using phased haplotypes as alleles. For both datasets, analyses were conducted by modelling admixture using a conditional autoregressive model (CAR). Preliminary analyses were conducted to assess model performance, with 20 000 steps (the first 5000 were discarded as burn-in) and 10 replicates for each K value (i.e. the number of clusters) between 2 and 10. The final analysis contained 100 replicates for each K value, with K = 2–10; each run consisted of 50 000 steps, with the first 20 000 discarded as burn-in. The spatial interaction parameter was initially kept at the default value (0.6), and the option to update it was activated. For both datasets, the model that best fitted the data was selected using the deviance information criterion (DIC). DIC values were averaged over the 100 replicates for each K value, and the most probable K value was selected as the one at which the average DIC reached a plateau. For the selected K value, the estimated admixture proportions of the 10 runs with the lowest DIC were averaged using CLUMPP 1.1.2.

To estimate the amount of variation attributable to differences among population groups, among populations within groups, and within populations, we performed a locus by locus analysis of molecular variance (AMOVA)^91^ on the microsatellite dataset using ARLEQUIN 3.5.1.3. Groups of populations were defined according to the results of the spatial clustering analysis with TESS. Admixed populations were attributed to the most represented cluster. The significance of variance components and fixation indices were tested using 1092 permutations.

### Phylogenetic and Bayesian phylogeographical analyses

Because no differences were detected by a partition-homogeneity test^92^ implemented in PAUP* 4.0B10, the two mtDNA fragments were combined into a unique haplotype using Concatenator 1.1.0. All of the subsequent analyses were conducted on the combined dataset.

Phylogenetic relationships between the mtDNA haplotypes were inferred using the maximum likelihood (ML) algorithm in PhyML 3.10. We used the default settings in PhyML for all of the parameters, except for the type of tree improvement (SPR and NNI) and the substitution model [HKY; selected using the Bayesian information criterion (BIC) in jModelTest 2.1.3^93^]. The robustness of the inferred tree topology was assessed using the non-parametric bootstrap method with 1000 pseudo-replicates. The estimated tree topology was then converted into a haplotype genealogy using Haplotype Viewer^94^. Genealogical relationships between the mtDNA haplotypes were also investigated using the statistical parsimony approach in TCS 1.21^95^, with a 95% cut-off criterion for a parsimonious connection. The statistical parsimony approach was also used to investigate genealogical relationships between haplotypes for each nDNA gene fragment. MEGA 6.0 was used to compute the net sequence divergence among the main haplogroups found.

The Bayesian procedure implemented in BEAST 1.8.1^96^ was used to estimate the divergence time among the main haplogroups that were identified by the previous phylogenetic analyses. For these analyses we only used the mtDNA dataset, because the nuclear gene fragments exhibited limited sequence variation (see Results). PartitionFinder 1.1 was used to select the optimal partitioning strategy and substitution models for each partition, using linked branch length options and forcing the software to choose only among the models implemented in BEAST. According to the BIC, the best scheme was the same for both genes: HKY for the first and second positions and TrN93 for the third position. This partition scheme was used for all subsequent analyses. A Bayesian skyline was chosen as a coalescent tree prior^97^. The molecular clock was calibrated by setting a normal prior for the treeModel.rootHeight parameter with a mean of 2.1 million years (standard deviation of 0.45), following the fossil-calibrated estimate of the TMRCA of *I*. *a*. *apuana* in Recuero *et al*.^41^. This calibration was preferred over a previous alternative^40^, which was based on hypothesized biogeographic scenaria and their influence on distantly related organisms (i.e. the potential influence of the Messinian salinity crisis on the divergence between species of the genus *Pleurodeles*). Preliminary analyses were run with an uncorrelated relaxed lognormal molecular clock model^98^; however, because the standard deviation (ucdl.stdev) was close to zero in the relaxed clock model, subsequent analyses were conducted with a strict clock model. Two final runs were performed, each with a Markov chain Monte Carlo (MCMC) length of 10 million generations, with sampling every 1000 generations. Traces were inspected using Tracer 1.6 to evaluate the ESS of the estimated parameters and the convergence between runs, after removing the first 10% of samples as burn-in. The two runs were combined using LogCombiner 1.8.1, and an annotated maximum clade credibility (MCC) tree was computed with TreeAnnotator 1.8.1 (in the same package).

The spatial and temporal diffusion patterns of *I*. *a*. *apuana* throughout its range were jointly estimated using a Bayesian Phylogeographic (BP) analysis in continuous space as implemented in BEAST^99^. To avoid any potential bias caused by the population structure^100^, we performed separate analyses with the same settings for each main haplogroup identified by the previous phylogenetic analyses. Geographical coordinates were provided for each individual after a slight perturbation of ±0.001 was applied to duplicate coordinates. BP analyses were run using a strict molecular clock model, the Bayesian skyline as a coalescent tree prior, MCMCs of length 90 million generations that were sampled every 9000 generations, and Cauchy as a model of spatial diffusion (see Lemey *et al*.^99^).

We visualized the spatial and temporal diffusion patterns by generating an annotated MCC tree with TreeAnnotator, which was then projected onto a geographical map using SPREAD 1.0.7. Finally, the full posterior of trees was analysed using the timeSlicer option in SPREAD, in order to estimate the geographical location of the MRCA of each lineage.

## Electronic supplementary material

Supplementary information

Supplementary information
