# Publisher Correction: Quaternary history, population genetic structure and diversity of the cold-adapted Alpine newt *Ichthyosaura alpestris* in peninsular Italy

**DOI:** 10.1038/s41598-018-21905-w

**Published:** 2018-03-06

**Authors:** Andrea Chiocchio, Roberta Bisconti, Mauro Zampiglia, Giuseppe Nascetti, Daniele Canestrelli

**Affiliations:** Dipartimento di Scienze Ecologiche e Biologiche, Università della Tuscia, Viale dell’Università s.n.c., I-01100 Viterbo, Italy

Correction to: *Scientific Reports* 10.1038/s41598-017-03116-x, published online 07 June 2017

The supplementary file “Bayesian phylogeographic recostruction.kmz” was omitted from the original version of this Article. This has been corrected in the HTML version of the Article; the PDF version was correct at time of publication.

