# Supplementary material for: Quaternary history, population genetic structure and diversity of the cold-adapted Alpine newt Ichthyosaura alpestris in peninsular Italy
Source: Sci Rep. 2017 Jun 7;7:2955. doi: 10.1038/s41598-017-03116-x (PMC5462806; doi:10.1038/s41598-017-03116-x)
Supplement: Supplementary file 1 — Supplementary information [file 41598_2017_3116_MOESM1_ESM.pdf]

Supplementary Information

Quaternary history, population genetic structure and diversity of the cold-adapted Alpine newt *Ichthyosaura alpestris* in peninsular Italy

Andrea Chiocchio[1], Roberta Bisconti[1*], Mauro Zampiglia[1], Giuseppe Nascetti[1], Daniele Canestrelli[1]

[1] Dipartimento di Scienze Ecologiche e Biologiche, Università della Tuscia, Viale dell'Università s.n.c., I-01100, Viterbo, Italy.

*Corresponding author: Roberta Bisconti, Dipartimento di Scienze Ecologiche e Biologiche, Università della Tuscia, Viale dell'Università s.n.c., I-01100, Viterbo, Italy, bisconti@unitus.it.

Table S1 Haplotypes found for each gene fragment analysed, and their abundance (in brackets) in each population studied of *Ichthyosaura alpestris* along the Italian peninsula.

Site	mtDNA	β-FIB	PDGFR	GH
1	AVIII(11)	AI(6), AII(6)	AII(9)	AI(18)
2	AI(10)	AI(14)	AI(1), AIV(4), AV(9)	AI(14)
3	AI(3), AIII(1)	AI(8)	AI(6), AIV(2)	AI(8)
4	AI(9)	AI(18)	AI(9), AIV(7)	AI(12), AII(2)
5	AI(4), AII(7)	AI(12)	AI(6)	AI(12)
6	AI(7)	AI(16)	AI(5), AIV(5)	AI(10), AII(3), AIV(1)
7	AI(8), AV(1)	AI(22)	AI(15), AIII(1), AIV(4)	AI(19), AII(1)
8	AI(10)	AI(16)	AI(11), AIV(5)	AI(16)
9	AVI(13), AVII(1)	AI(13), AIV(2), B1(5), BII(2)	AI(28)	AI(12), CI(4)
10	AVI(12), BII(2)	AI(13), AIII(1), BII(2)	AI(18)	AI(9), BI(5)
11	BI(8), BVI(1)	AI(4), BI(4), BII(2)	AI(12)	AI(6), BI(4)
12	BI(9)	AI(4), BI(4), BII(2)	AI(10)	AI(5), AIII(2), BI(1)
13	BI(3), BIV(2), BV(1), BIII(6)	AI(6), BI(6)	AI(16)	AI(11), BI(3)
14	BII(10)	BII(8), AI(2)	AI(10)	AI(3), AV(1), BI(7), BII(1)
15	BI(10)	AI(10)	AI(14)	AI(5), BI(10), BII(1)

Table S2 Allele frequencies at the polymorphic microsatellite loci studied among 15 populations of *Ichthyosaura alpestris* in peninsular Italy.

	**POPULATION**														
**LOCUS/ ALLELE**	**1**	**2**	**3**	**4**	**5**	**6**	**7**	**8**	**9**	**10**	**11**	**12**	**13**	**14**	**15**
**Copta1**															
**229**	1.00	1.00	1.00	1.00	1.00	1.00	1.00	1.00	0.63	0.86	0.57	0.28	0.23	---	0.33
**231**	---	---	---	---	---	---	---	---	0.37	0.14	0.43	0.72	0.77	1.00	0.67
**Copta3**															
**162**	---	---	---	---	---	---	---	---	0.03	---	---	---	---	---	---
**168**	---	---	---	---	---	---	---	0.04	---	---	---	---	---	---	---
**170**	---	---	---	---	---	---	0.04	---	0.06	---	---	---	---	---	---
**172**	1.00	1.00	1.00	1.00	1.00	1.00	0.96	0.96	0.91	1.00	1.00	1.00	0.92	1.00	1.00
**174**	---	---	---	---	---	---	---	---	---	---	---	---	0.08	---	---
**Copta8**															
**165**	---	0.71	0.70	0.79	0.81	0.72	0.92	0.62	0.56	0.75	1.00	1.00	1.00	1.00	1.00
**167**	---	0.29	0.30	0.21	0.19	0.28	0.04	0.35	0.34	0.25	---	---	---	---	---
**169**	0.68	---	---	---	---	---	0.04	0.04	0.09	---	---	---	---	---	---
**171**	0.32	---	---	---	---	---	---	---	---	---	---	---	---	---	---
**Copta9**															
**218**	---	---	---	---	---	---	---	---	---	---	0.04	0.05	0.04	---	---
**222**	---	---	---	0.17	---	---	---	---	---	---	0.08	---	---	0.12	---
**226**	0.04	0.04	---	0.04	---	---	---	0.04	---	0.11	0.19	0.25	0.08	0.23	---
**230**	0.04	0.11	---	0.04	0.15	---	0.22	0.04	---	0.21	0.23	0.20	0.08	0.31	0.50
**234**	0.19	---	0.17	0.08	0.04	---	0.06	---	0.30	0.21	0.08	0.35	0.33	0.35	---
**238**	0.08	0.14	---	0.04	0.15	0.15	---	0.27	0.20	---	0.19	0.10	0.17	---	0.05
**242**	---	0.04	0.25	0.33	0.04	0.05	0.11	---	0.10	0.25	0.04	0.05	0.13	---	0.40
**246**	0.23	---	---	0.04	---	0.10	0.11	---	0.10	---	---	---	0.08	---	---
**250**	---	---	---	---	0.08	0.35	0.11	0.19	0.07	---	0.15	---	0.04	---	0.05
**254**	---	0.21	0.33	---	0.12	0.10	0.06	0.35	---	---	---	---	---	---	---
**258**	0.04	---	0.08	---	0.04	---	0.17	0.12	---	0.07	---	---	---	---	---
**262**	0.19	0.04	---	0.08	0.23	0.10	0.06	---	---	0.11	---	---	0.04	---	---
**266**	0.12	---	---	0.04	0.04	---	---	---	---	---	---	---	---	---	---
**270**	---	0.07	---	---	---	---	0.06	---	0.23	---	---	---	---	---	---
**274**	---	0.04	---	0.13	---	0.15	---	---	---	0.04	---	---	---	---	---
**278**	---	0.11	0.08	---	---	---	---	---	---	---	---	---	---	---	---
**282**	0.08	0.14	---	---	0.12	---	---	---	---	---	---	---	---	---	---
**286**	---	0.04	0.08	---	---	---	0.06	---	---	---	---	---	---	---	---
**294**	---	0.04	---	---	---	---	---	---	---	---	---	---	---	---	---
**Copta13**															
**199**	---	0.63	---	---	0.77	0.06	0.04	---	---	0.04	---	---	---	---	---
**203**	1.00	0.38	1.00	1.00	0.23	0.94	0.96	1.00	1.00	0.96	1.00	1.00	1.00	1.00	1.00
**Ta3Ca8**															
**125**	---	---	---	---	---	---	---	---	---	---	0.19	---	0.08	---	---
**131**	---	---	---	---	0.17	0.05	---	---	0.09	0.08	0.35	0.11	0.25	0.05	---
**135**	---	---	---	---	---	---	---	---	---	---	---	---	0.08	---	---
**141**	---	---	0.25	---	0.04	0.10	0.09	0.15	0.38	0.42	0.15	0.17	0.33	0.85	1.00
**145**	---	---	---	---	---	0.05	---	---	---	---	---	---	---	---	---
**147**	---	---	0.25	0.09	0.21	0.30	0.68	---	0.22	0.17	0.19	0.72	0.25	0.05	---
**151**	0.15	1.00	0.50	0.91	0.58	0.50	0.23	0.85	0.31	0.33	0.12	---	---	0.05	---
**153**	0.85	---	---	---	---	---	---	---	---	---	---	---	---	---	---
**Ta3Caga2**															
**132**	---	---	0.07	---	---	---	---	---	---	---	---	---	---	---	---
**136**	1.00	1.00	0.93	1.00	1.00	1.00	1.00	1.00	0.94	0.54	0.54	0.85	0.92	0.46	1.00
**160**	---	---	---	---	---	---	---	---	0.06	0.46	0.46	0.15	0.08	0.54	---
